# Comparison of glucose variability measures

**DOI:** 10.1186/cc9827

**Published:** 2011-03-11

**Authors:** M Bhattacharyya, S Todi

**Affiliations:** 1 AMRI Hospitals, Kolkata, India

## Introduction

Glycemic excursion or glucose variability (GV) is associated with short-term ICU mortality. There is a heterogeneity among studies in using measures of GV. The objective of this study was to compare different formulas used to assess GV in predicting mortality.

## Methods

The study was done in a 45-bed medical-surgical unit. All patients admitted to the ITU and with four or more blood glucose (BG) readings were included from January 2009 to November 2009. Sugar control was protocolised with a target CBG of ≤150 mg/dl. Glucose was measured from central laboratory or point-of-care checking at an interval of 6 hours or when required. From the prospectively collected glucose values, different measures of glycemic variability have been calculated and compared among themselves. We used standard deviation (SD), glycemic lability index (GLI), maximum glucose change (MGC), mean amplitude of glucose excursion (MAGE), and average daily risk range (ADRR) as measures of GV.

## Results

**Figure 1:**
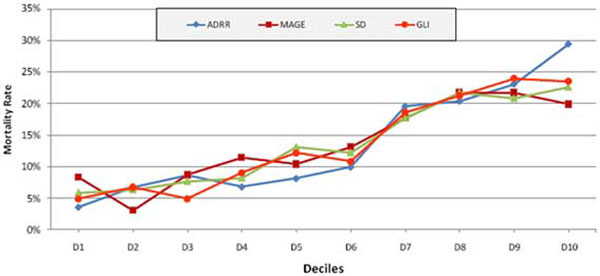
**Comparison of deciles of different GV formulas with mortality**.

**Figure 2:**
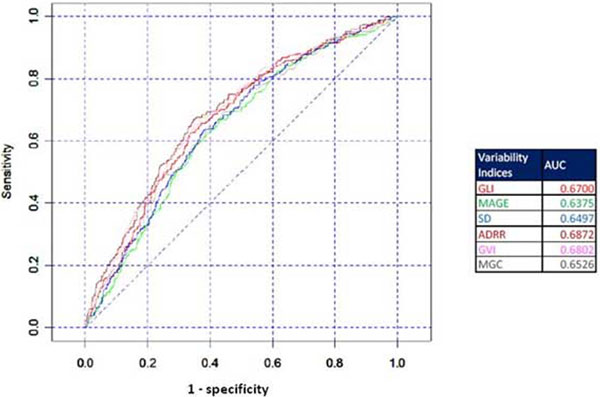
**ROC of formulas**.

A total of 11,335 blood sugar records were analyzed from 2,208 patients during this time. Mean age of the study population was 61 (SD ± 16.71). In total, 58.96% were male and 77.8% were medical admissions. The mean APACHE IV score was 56.9. All the variables of GV could predict mortality with equal power. See Figure 1 and 2.

## Conclusions

All of the GV measures have almost the same prediction power. Any one measure can be used as a quality indicator of GV in an ICU.

